# DNA fragmentation and multifaceted toxicity induced by high-dose vanadium exposure determined by the bioindicator *Allium* test

**DOI:** 10.1038/s41598-023-35783-4

**Published:** 2023-05-25

**Authors:** Mehmet Kaya, Kültiğin Çavuşoğlu, Emine Yalçin, Ali Acar

**Affiliations:** 1 Institute of Science, Giresun University, Giresun, Turkey; 2 Department of Biology, Faculty of Science and Art, Giresun University, Giresun, Turkey; 3 Department of Medical Services and Techniques, Vocational School of Health Services, Giresun University, Giresun, Turkey

**Keywords:** Cell biology, Genetics

## Abstract

In this study, the toxicity of vanadium (VCI_3_) in *Allium cepa* L. was studied. Germination-related parameters, mitotic index (MI), catalase (CAT) activity, chromosomal abnormalities (CAs), malondialdehyde (MDA) level, micronucleus (MN) frequency and superoxide dismutase (SOD) activity were investigated. The effects of VCI_3_ exposure on the DNA of meristem cells were investigated with the help of comet assay, and the relationships between physiological, cytogenetic and biochemical parameters were revealed by correlation and PCA analyses. *A. cepa* bulbs were germinated with different concentrations of VCI_3_ for 72 h. As a result, the maximum germination (100%), root elongation (10.4 cm) and weight gain (6.85 g) were determined in the control. VCI_3_ treatment caused significant decreases in all tested germination-related parameters compared to the control. The highest percentage of MI (8.62%) was also observed in the control. No CAs were found in the control, except for a few sticky chromosomes and unequal distribution of chromatin (*p* > 0.05). VCI_3_ treatment caused significant decreases in MI and increases in the frequencies of CAs and MN, depending on the dose. Similarly, the comet assay showed that DNA damage scores increased with increasing VCI_3_ doses. The lowest root MDA (6.50 µM/g) level and SOD (36.7 U/mg) and CAT (0.82 OD_240nm_min/g) activities were also measured in the control. VCI_3_ treatment caused significant increases in root MDA levels and antioxidant enzyme activities. Besides, VCI_3_ treatment induced anatomical damages such as flattened cell nucleus, epidermis cell damage, binuclear cell, thickening in the cortex cell wall, giant cell nucleus, damages in cortex cell and unclear vascular tissue. All examined parameters showed significant negative or positive correlations with each other. PCA analysis confirmed the relations of investigated parameters and VCI_3_ exposure.

## Introduction

Elements that are necessary in very low amounts for the development and continuation of physiological activities of organisms are called trace elements. Trace elements are involved in the activation of enzymes and proteins, which make up 0.02% of living weight and are involved in organism development and physiological activities^1^. They are involved as electron acceptors/donors in redox reactions and are responsible for the stability of biological molecules. In deficiency, enzyme activity, nutrition and growth in plants are adversely affected, while excessive intake can cause toxicity in human animals and plants^2^. Vanadium is in the group of possible trace elements together with fluorine, chromium, selenium and nickel. Vanadium, which has a melting point of 1910 °C, is also defined as a refractory metal. It has good structural strength and also shows good resistance to corrosion. It is also used in the manufacture of ceramics, in the manufacture of coatings for energy storage equipment, in the manufacture of microelectronic devices and smart windows. Vanadium is also used as a component of batteries^3,4^. Vanadium is commonly found in minerals, sedimentary and igneous rocks. Vanadium can be found in the environment in various chemical forms. Vanadium with various valence states (+ 5, + 4, + 3, + 2, 0, − 1 and − 3) is most commonly found in vanadium (IV) or vanadium (V) forms^5^. In compound form, it is possible to encounter organic or inorganic compounds. Inorganic vanadium compounds can be classified as chlorides, sulfates, oxides, vanadates, carbides and nitrides. The biochemical and toxic properties of natural chemical forms and compound forms may vary according to the biotic factors of the environment^5,6^. Vanadium is an element that can be found in the air, soil, plants, and water. Its average amount in the earth's crust is about 138 ppm. The average concentration of V found in soils around the world is reported to be 108 mg/kg, but the V values investigated and reported in studies are generally below this average^6,7^. The vanadium concentration in aquatic environments is affected by many factors, but it is generally between 0.2 and 100 µg/L in fresh water and 0.2–29 µg/L in seawater^8^. Although the limit values defined for drinking water, soil or spring water vary, the limit value of vanadium in drinking water is generally determined as 50 μg/L^9^.

Vanadium is thought to be a micronutrient for mammals, including humans. However, its role in this matter has not yet been fully elucidated. However, trace amounts of vanadium are thought to be essential for normal cell growth. People may be exposed to vanadium occupationally in the processing of vanadium sources, the manufacture of products containing vanadium, and in some metallurgical plants^4–6^. In humans, chronic exposure can cause skin, respiratory tract irritation, greenish-black discoloration of the tongue, bronchial and tracheal inflammation, eye and skin irritation and systemic poisoning^6,8^. On the other hand, vanadium-based therapeutic drugs are also used for the treatment of many diseases. In addition, vanadium compounds have recently been preferred in chemotherapy applications as anti-cancer agents due to their strong cytotoxicity and relatively low systemic toxicity^10^. Vanadium is associated with the redox reactions of nitrogenase enzymes in some diazotrophs, specific haloperoxidases found in some organisms. Vanadium also acts as the active site of the vanadium bromoperoxidase enzymes of some oceanic algae^5^. Vanadium is essential for plants in low amounts for chlorophyll synthesis, K consumption and N assimilation. In most plants, low concentrations of V exert a stimulating effect on plant growth and development. It also acts as a redox catalyst in photosystems I and II in the electron transport system in plants. High vanadium concentrations cause chlorosis in plants, limit growth, reduce photosynthesis, disrupt mineral homeostasis and promote oxidative damage^7^. There are studies in the literature reporting the toxic effects of vanadium on plants. It has been reported that exposure to 120 μM vanadium inhibited shoot growth in *Triticale*^11^, at doses of 1 mM and 660–120 mg/L it inhibited root growth in rice and chickpea^12,13^ and 25–100 µg/L vanadium-induced anatomical damages in *Allium cepa*^14^. In the literature, limited parameters were used in studies investigating the toxic effects of vanadium on *A. cepa*, while physiological, biochemical, anatomical, cytological and genetic parameters were used in this study. And also in this study, a multifaceted toxicity profile and possible toxicity mechanisms were revealed by correlating the findings obtained in each parameter with each other. *Allium* test is one of the simple direct methods used to determine the effects of mutagens or potentially toxic substances and an excellent in-vivo model used to predict possible abnormalities in the eukaryotic DNA. The *Allium* test exhibits good correlation and high sensitivity with mammalian tests, and the same sensitivity with test systems using human lymphocytes and algae^15,16^. Advantages such as easy availability, rapid results, easy application and low cost increase its usability in toxicity tests. The fact that *A. cepa* is a vascular plant makes this test a useful test model for determining the cytogenetic effects of environmental pollutants. The most important advantage is that it allows the determination of chromosomal abnormalities (CAs), micronucleus (MN) and mitotic index (MI) in one go with the prepared cytogenetic preparations^17,18^.

## Material and methods

### Test material

*A. cepa* bulbs (*2n* = 16), which is a eukaryotic indicator organism, were preferred as test material. Bulbs were obtained from a local market in Giresun.

### Test chemical

Vanadium (III) chloride (VCI_3_-CAS No: 7718-98-1-25 G), a product of Merck company, was used as test chemical. VCI_3_ doses were determined according to EC_50_. The EC_50_ concentration for VCI_3_ was calculated as 100 µg/L. Along with the EC_50_ dose, a dose of 200 µg/L, which is twice the EC_50_, and 50 µg/L, which is half of it, were preferred.

### Experimental process

In the study, healthy and equal-sized bulbs were used and their outer shells were removed before the application. The bulbs were divided into 4 groups and control bulbs were treated with tap water. Vanadium application was used at concentrations determined according to the EC_50_ value and for this purpose, the bulbs in the application group were germinated with VCI_3_ at doses of 50 µg/L, 100 µg/L and 200 µg/L. The bulbs of the control and the treatment groups were germinated at 24 °C for 72 h. At the end of the period, the bulbs and root tips collected from each group were used for experimental analysis, measurement and observations^19^. Experimental research on plant samples, including the supply of plant material, complies with institutional, national and international guidelines and legislation. In this study, the toxic effects and possible mechanisms of VCI_3_ were elucidated by using different parameters.

### Physiological parameter measurements

Root growth was assessed by determining the radicle length and the weight gain was determined by measuring the weights of each bulb before and after the application. The germination percentage (GP) was calculated using Eq. (1)^19,20^. In the germination percentage test 50 bulbs; in root length and weight gain analysis 10 bulbs were tested.

1 $${\text{GP }}\left( \% \right) \, = \, \left[ {{\text{germinated}}\,{\text{bulb}}\,{\text{number}}} \right]/\left[ {{\text{total}}\,{\text{bulb}}\,{\text{number}}} \right] \, \times { 1}00.$$

### Cytogenetic parameter observations and counts

Mitotic slides were prepared from root tip samples to observe CAs and MN and to examine MI rates. After germination, root tip samples of each group were collected and subjected to routine fixation, hydrolysis and staining processes. Acetocarmine was used for staining mitotic chromosomes in root tip cells. Mitotic slides were examined under a research microscope^19,21^. Observations and counts of CAs and MN were performed by two different observers to ensure their accuracy. The criteria determined by Fenech et al.^22^ were taken into account in the determination of MN formations. 1000 cells were analyzed for MN and CAs analysis. MI was calculated with Eq. (2) and calculated by analyzing 10,000 cells in each group.

2 $${\text{MI }}\left( \% \right) \, = \, [{\text{Number}}\,{\text{of}}\,{\text{cells}}\,{\text{in}}\,{\text{mitosis}}\left] / \right[{\text{total}}\,{\text{counted}}\,{\text{cell}}\,{\text{number}}] \, \times { 1}00.$$

### Comet test

Comet analysis was applied according to the protocol suggested by Chakraborty et al.^23^. Root tip samples were crushed gently in 400 mM Tris-buffer for the isolation of nuclei to obtain nuclear suspension. Slides immersed in 1% NMPA solution were dried at room temperature overnight. 40 µL of nuclear suspension and 40 µL of 1% LMPA from each group were gently mixed and added to each slide. A coverslip was placed over the mixture to obtain a uniform layer. After solidification, the coverslip was removed and a layer of 80 µL 0.5% LMPA was formed on the surface. Slides containing the nuclear suspension were transferred to gel electrophoresis tank with Na_2_EDTA and NaOH (pH > 13). After waiting for 15 min, electrophoresis was performed at 4 °C and 0.7 V/cm (20 V and 300 mA) for 20 min. Slides rinsed with tris-buffer for neutralization were stained with ethidium bromide (20 μg/mL) for 5 min and examined under fluorescence microscopy. Comet scores (tail length) were analyzed with the help of Comet Assay Software (CASP-version 1.2.3b)^24^. A total of 1000 cells per group, 100 in each bulb, were analyzed for DNA damage. Comet analyzes were repeated twice with CASP on the slides prepared for each group. Cells were analyzed for five categories, from zero to four, according to varying tail DNA lengths as stated by Collins^25^. Total DNA damage per group was calculated using Eq. (3).

3 $$Arbitrary \,unit= {\sum }_{i=0}^{4}Ni \times i$$

Ni: the number of cells in i degree, i: degree of damage (0, 1, 2, 3, 4).

### Biochemical parameter measurements

MDA levels were measured according to the procedure recommended by Unyayar et al.^26^. 0.5 g of the sample was homogenized with 5% trichloroacetic acid. 0.5% thiobarbituric acid was added to the supernatant obtained after centrifugation and then incubated at 96 °C in 20% trichloroacetic acid. The absorbance of the supernatant obtained by centrifugation at the end of the incubation was measured at 532 nm^19^. The root tips of each group were collected and extracted for enzyme activities. For this purpose, 0.5 g of sample was homogenized and the supernatant was used for biochemical analysis^19,27^. SOD enzyme activities (U/mg FW) were measured according to the procedure proposed by Beauchamp and Fridovich^28^. CAT activity (OD_240nm_min/g FW) was measured according to the method developed by Beers and Sizer^29^. All analyzes were performed in triplicate to determine the levels of SOD, CAT and MDA.

### Anatomical examinations

Cross-sections of root samples from each group were taken to investigate the effects of vanadium exposure on root anatomy. Root samples were washed three times consecutively with distilled water to remove surface residues then cross-sections were taken from root tips collected from bulbs belonging to each group. Sections placed between the slide and the coverslip were stained with 5% methylene blue and made into a fixed preparation with Canadian balm. Anatomical structures were examined with the IM-450 TI model research microscope^30^.

### Statistical analyzes

SPSS Statistics 22 (IBM SPSS, Turkey) package program was used for statistical analysis. Statistical significance between data as mean ± standard deviation (SD) was determined by one-way analysis of variance, “One-way Anova” and “Duncan” tests. P values less than 0.05 were considered statistically significant. Correlation and principal component analyzes (PCA) were performed with Rstudio v 1.4.1106 program^31^. Pearson correlation analysis (bilateral) and graphs were obtained with hmisc and corrplot packages^32^. PCA was performed according to physiological, biochemical and genetic parameters, which are different toxicity biomarkers tested. PCA analysis was performed using the factoMineR^33^ and factoextra^34^ packages available in RStudio.

## Results and discussion

### Physiological findings

The effects of VCI_3_ application on germination-related parameters are shown in Table 1. A normal germination process and the highest germination parameters were obtained in the control. Physiological parameters tested in this study regressed in the groups treated with VCI_3_. The most significant regression was detected in the group treated with 200 µg/L VCI_3_. In Group IV, germination rate, root length and weight gain decreased by 28%, 2.6 times and 2.3 times, respectively compared to control. Our results are similar to literature studies investigating the physiological effects of vanadium and vanadium compounds. Vachirapatama et al.^35^ reported a decrease in stem length, leaf number, leaf, stem and root dry weight as the vanadium concentration increased in green mustard and tomato plants exposed to ammonium meta vanadate (*NH*_*4*_*VO*_*3*_). Macar et al.^14^ reported that 25, 50 and 100 µg/L vanadyl sulfate pentahydrate (VOSO_4_· 5H_2_O) exposure caused significant reductions in germination-related parameters in *A. cepa* bulbs. Aihemaiti et al.^36^ reported a 50% reduction in root length of *Setaria viridis* grown in soil containing 40–55.8 mg/L vanadium.

**Table 1:** The effects of VCI_3_ on germination-related parameters.

Groups	Germination percentage (%)	Root length (cm)	Weight gain (g)	Initial weight (g)	Final weight (g)
Control	100	10.4 ± 1.16^a^	+ 6.85^a^	9.52 ± 0.90	16.37 ± 1.29
50 µg/L VCI_3_	91	8.80 ± 0.97^b^	+ 5.40^b^	9.58 ± 0.92	14.98 ± 1.21
100 µg/LVCI_3_	83	7.50 ± 0.85^c^	+ 4.12^c^	9.45 ± 0.87	13.57 ± 1.18
200 µg/L VCI_3_	72	4.00 ± 0.54^d^	+ 2.97^d^	9.66 ± 0.95	12.63 ± 1.12

The mean ± SD values shown with different letters (a–d) in the same column are statistically significant.

Abnormalities observed in germination parameters as a result of VCI_3_ exposure may be associated with the decrease in nutrient intake of root, damages in the root structure and regression in mitotic cell division. The biotransformation process including reduction reactions takes place in the uptake of vanadium by plant roots, and aldehyde, ketone, catechol, olefin and sulphide groups are oxidized in these regions. These reactions provide a strong attachment and immobilization of vanadium to root tissues. In this way, vanadium remaining in the root for a long time causes serious toxic effects^5^. Delays and abnormalities in root growth also reported in literature studies confirm this claim. Vachirapatama et al.^35^ determined that high doses of vanadium damaged the structure of tomato roots and prevented them from working efficiently. In our study, structural abnormalities such as epidermis and cortex cell damage were observed in *A. cepa.* The same researchers determined that vanadium inhibits plasma membrane ATPase activity, which is known to have an important role in the uptake of nutrients by plant cells, and fructose 2,6-biphosphatase, which is involved in photosynthesis. Also heavy metal ions inhibit root growth by slowing root meristem cell division^19,37^. In our study, a decrease in MI values of root meristem cells was observed due to the increase in VCI_3_ dose.

### Genotoxicity findings

**Figure 1:**
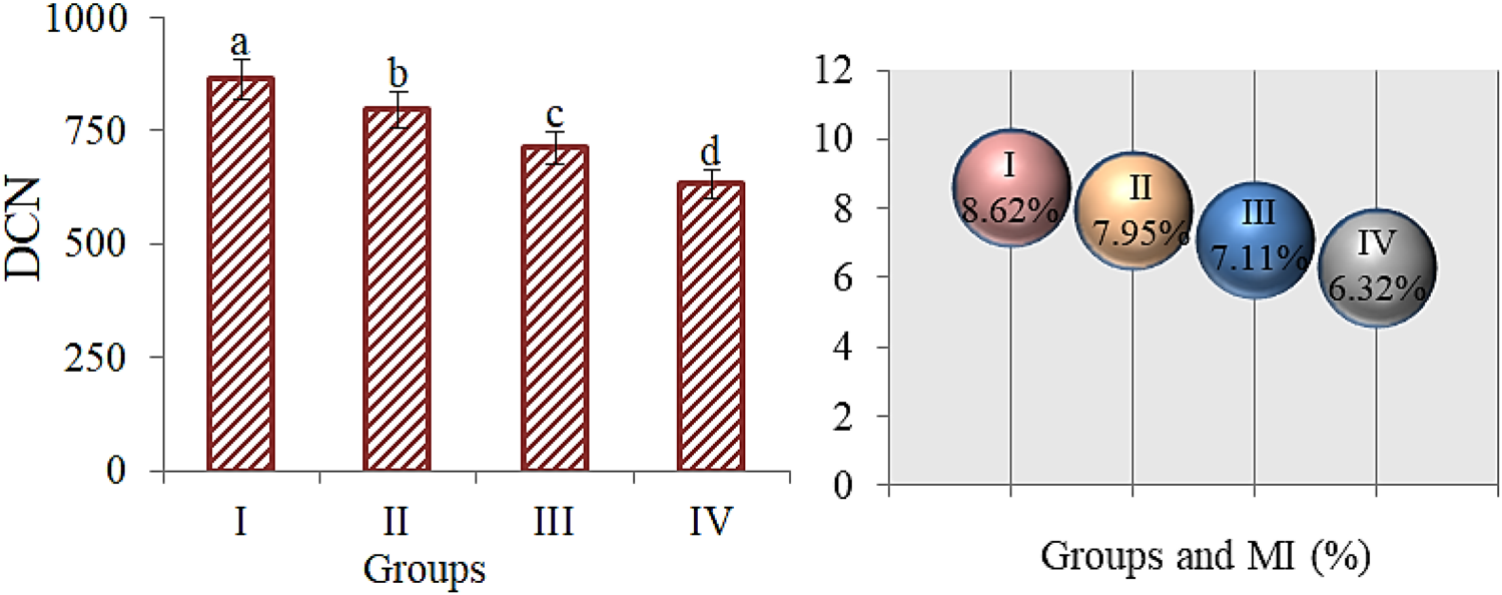
The effects of VCI_3_ on MI (%) and dividing cell number (DCN). Different letters^(a–d)^ on the figure are statistically significant at p < 0.05.

**Figure 2:**
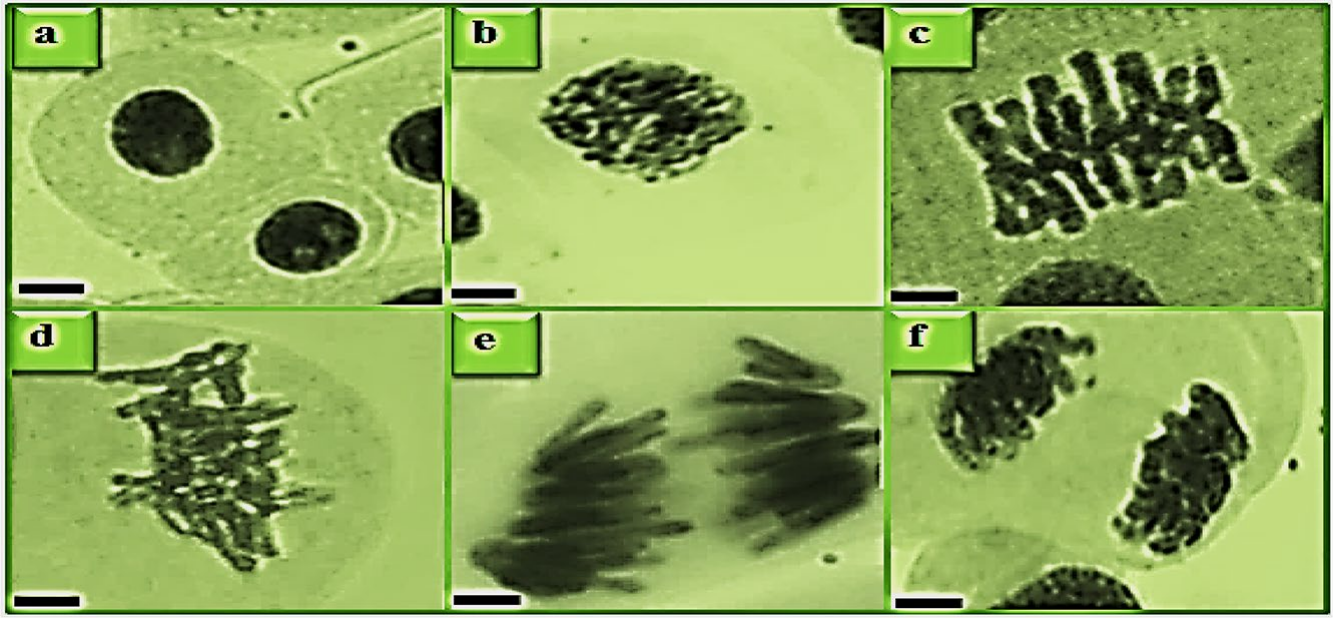
Stages of mitosis in control. Interphase (**a**), prophase (**b**), metaphase (**c**), early anaphase (**d**), late anaphase (**e**), telophase (**f**). Bar: 10 µm.

**Figure 3:**
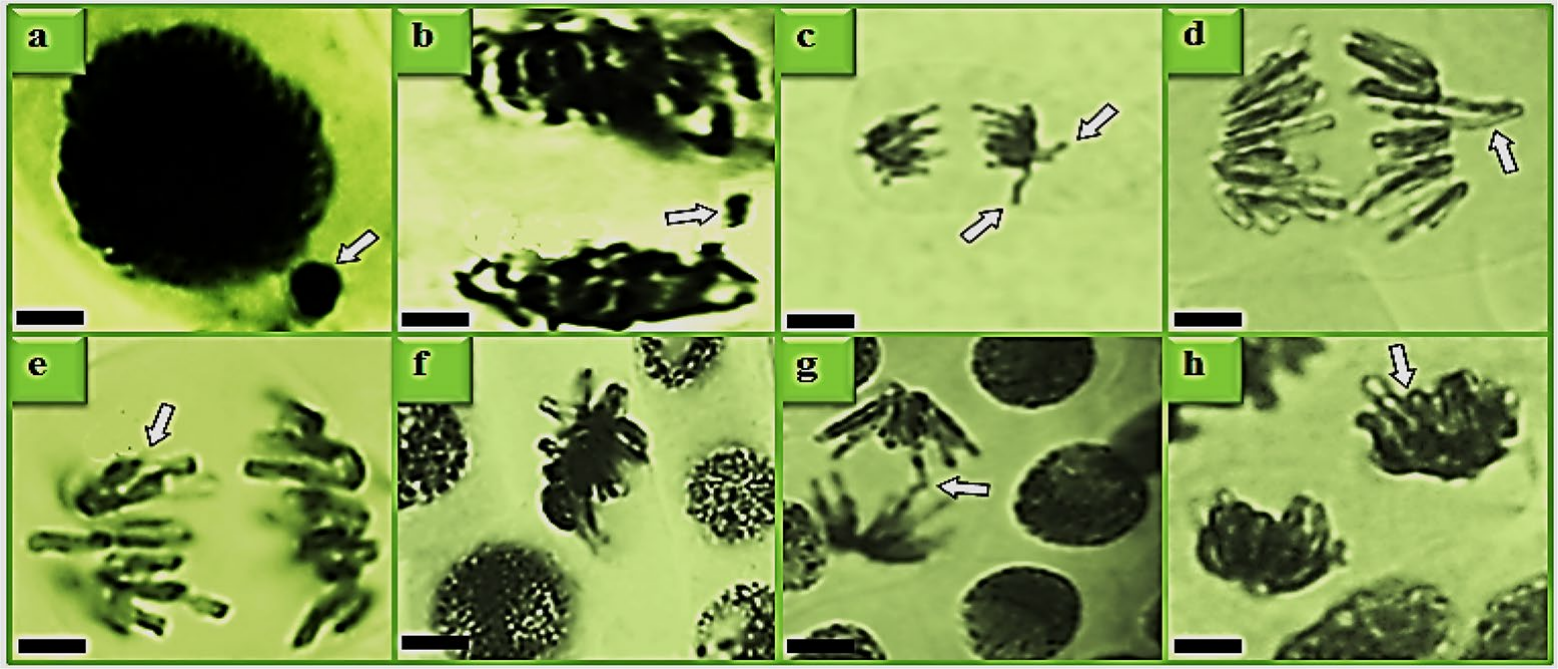
CAs induced by VCI_3_ toxicity. MN (**a**), fragment (**b**), vagrant chromosome (**c**,**d**), unequal distribution of chromatin (**e**), sticky chromosome (**f**), bridge (**g**), reverse polarization (**h**). Bar: 10 µm.

Genotoxicity induced by VCI_3_ exposure is shown in Table 2. The highest MI, lowest MN frequency and CAs levels were obtained in the control. VCI_3_ exposure caused a decrease in MI and an increase in MN and CAs numbers (*p* < 0.05). Vanadium administration caused serious reductions in MI rates, confirming the regressions in physiological parameters (Fig. 1). Among the VCI_3_ application groups, the highest decrease in MI value and the highest increase in the frequency of MN and the number of CAs were determined in 200 µg/L VCI_3_-treated group. In Group IV, MI decreased by 2.3%, while the frequency of MN increased 31 times and fragment damage, the most observed CAs, increased 29 times compared to the control. The stages of mitosis in the control are given in Fig. 2 and the different types of CAs formations induced by vanadium application are given in Fig. 3. Among CAs abnormalities, a high rate of fragments was observed, while reverse polarization was observed at the lowest level.

**Table 2:** VCI_3_ effects on MN and CAs frequencies.

Damages	Control	50 µg/L VCI_3_	100 µg/L VCI_3_	200 µg/L VCI_3_
MN	N.O^d^	8.70 ± 0.84^c^	14.2 ± 1.27^b^	30.6 ± 2.48^a^
FRG	N.O^d^	7.60 ± 0.78^c^	12.9 ± 1.22^b^	28.5 ± 2.36^a^
VC	N.O.^d^	6.80 ± 0.74^c^	11.7 ± 1.18^b^	26.4 ± 2.28^a^
UDC	0.20 ± 0.32^d^	5.90 ± 0.69^c^	10.3 ± 1.05^b^	23.8 ± 2.15^a^
SC	0.16 ± 0.42^d^	4.80 ± 0.62^c^	9.50 ± 0.96^b^	20.3 ± 1.97^a^
B	N.O^d^	3.50 ± 0.56^c^	8.40 ± 0.88^b^	17.5 ± 1.78^a^
RP	N.O^d^	2.90 ± 0.49^c^	5.80 ± 0.64^b^	13.7 ± 1.45^a^

The mean ± SD values shown with different letters (a–d) in the same column are statistically significant. *FRG* fragment, *VC* vagrant chromosome, *B* bridge, *UDC* unequal distribution of chromatin, *SC* sticky chromosome, *RP* reverse polarization, *MN*: micronucleus, *N.O* not observed.

Our results are compatible with the findings of literature studies investigating the genotoxicity induced by vanadium and vanadium compounds. Marcano et al.^38^ did not detect any change in MI values of *A. cepa* exposed to vanadium solutions, while they found an increase in the frequencies of CAs. Roychoudhury^5^ reported that meristematic cells of *Lens culinaris* (*lentil*) seeds exposed to vanadium stress had various CAs such as break, bridge, sticky chromosome and ring chromosome. On the other hand, studies of vanadium-induced genotoxicity have focused more on cultured human peripheral lymphocyte cells. Rodriguez-Mercado et al.^39^ reported that vanadium (IV) tetraoxide (V_2_O_4_) induced a decrease in mitotic levels of human lymphocyte and leukocyte cells in culture and a significant increase in the frequency of CAs. In another study by Rodríguez-Mercado et al.^40^, structural CAs such as break, acentric fragment, dicentric and gap, and a decrease in MI were detected in human peripheral lymphocyte cells exposed to different compounds of vanadium.

The alteration in MI value and the induction of MN and CAs formations by VCI_3_ treatment can be explained by the damage in DNA and microtubules (α-tubulin and β-tubulin proteins) due to induced oxidative stress. Because Çanlı^41^ reported that high vanadium concentrations significantly inhibited cell division. Similarly, Rodríguez-Mercado et al.^38^ associated the decrease in MI as a result of vanadium exposure with the decrease in cell proliferation rate. Imtiyaz et al.^13^ determined that vanadium stress causes DNA fragmentation by changing DNA integrity. Explaining the possible mechanisms of vanadium genotoxicity, Rodríguez-Mercado et al.^40^ reported that the vanadium ion can bind to DNA by interacting with the N-7 atoms of guanine and adenine bases and the PO_2_ groups in the backbone, or to form hydroxyl radicals (**·**OH) by reacting with hydrogen peroxide. They also stated that the vanadium ion produces **·**OH radicals, especially through fenton-type reactions, and these radicals can cause single and double-chain breaks in the DNA skeleton, sugars and bases by damaging the phosphate groups in DNA.

### Comet assay findings

**Figure 4:**
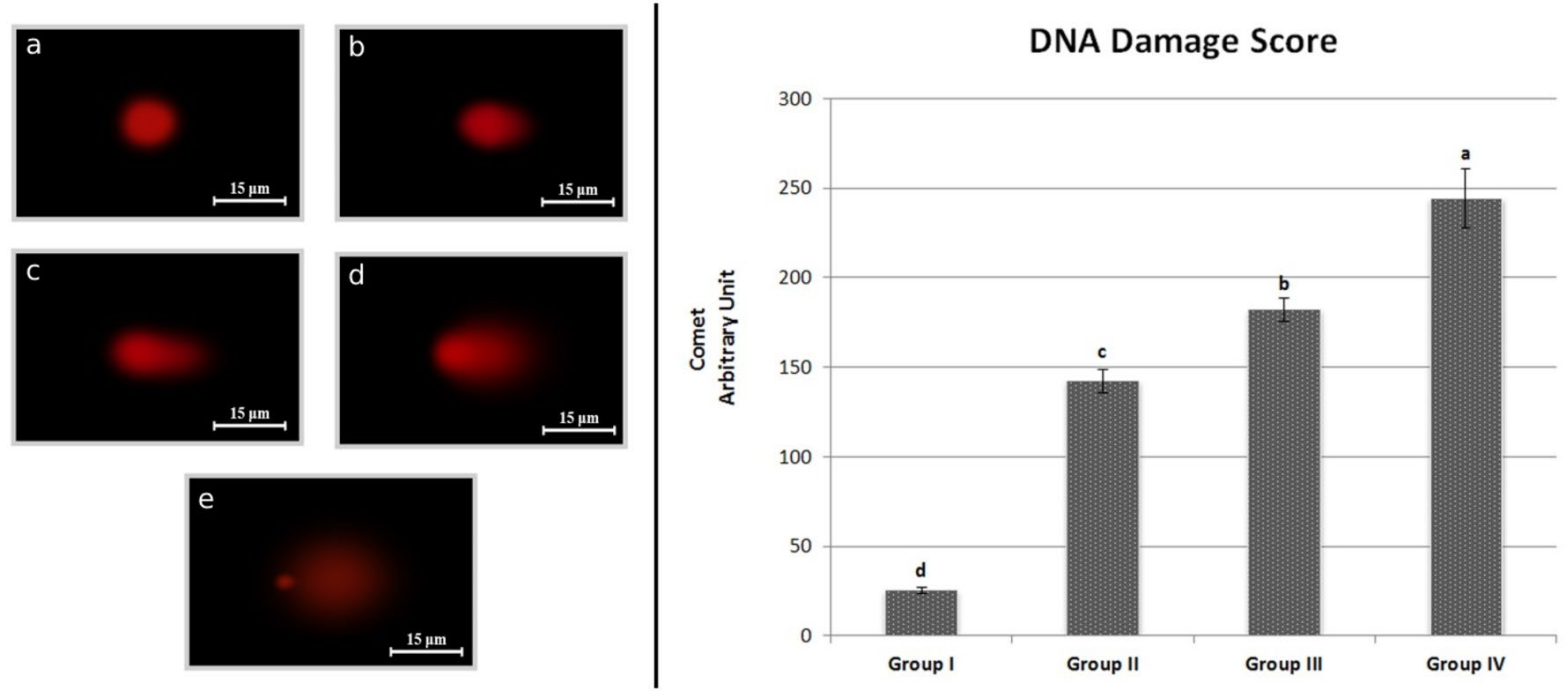
Effect of VCI_3_ on DNA fragmentation. (**e**) Extreme damage, (**d**) high damage, (**c**) moderate damage, (**b**) low damage, (**a**) no damage. Data are given as mean ± SEM. Different letters^(a–d)^ on the figure are statistically significant at p < 0.05.

The effects of VCI_3_ exposure on DNA fragmentation are shown in Fig. 4. The DNA damage score was 25.50 ± 1.63, 142.30 ± 6.54, 182.10 ± 6.73 and 244.30 ± 16.30 in control, Group II, III and IV, respectively. It was observed that the DNA damage score increased with increasing VCI_3_ doses. This shows that the frequency of DNA fragmentation and double-strand breaks increases depending on the increase in VCI_3_ dose. In addition, the differences between the DNA damage scores observed between the groups were found to be statistically significant (*p* < 0.05). Although there is no study investigating the effect of VCI_3_ on DNA damage in the plant test material, there are studies investigating the DNA damage induced in *A. cepa* L. with the comet assay. Seth et al.^42^ investigated the effects of cadmium on DNA damage in *A. cepa* L. with comet assay and found that DNA damage increased with increasing exposure dose, and this test material was a suitable model for detecting DNA damage with the comet assay. Yıldız et al.^43^ determined by the comet test that cobalt chloride and copper sulfate exposure caused an increase in DNA damage with increasing doses. In addition, our results are comparable to the literature studies reporting that vanadium induces DNA damage in different organisms. Leopardi et al.^44^ reported that vanadium exposure caused double chain breaks of splenocyte cells of mice, resulting in an increase in comet tail lengths. Rivas-Garcia et al.^45^ observed that exposure to vanadium in their comet test analysis caused an increase in DNA tail moment by promoting DNA damage in the HepG2 cell line.

### Biochemical findings

**Figure 5:**
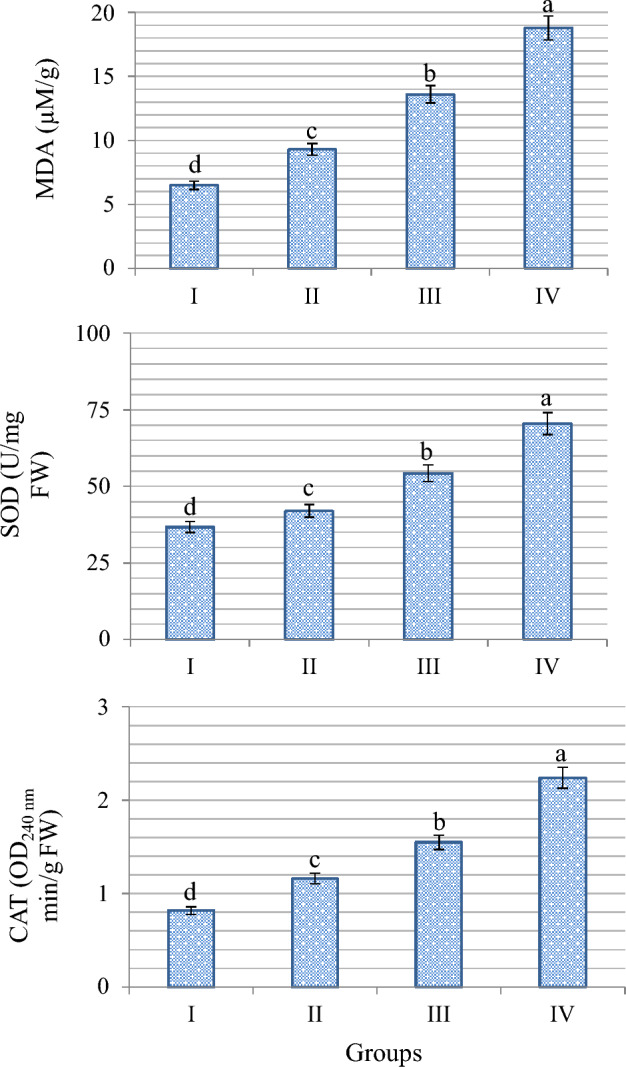
Biochemical effects of VCI_3_ toxicity. Different letters^(a–d)^ on the figure are statistically significant at p < 0.05.

The effects of VCI_3_ treatment on antioxidant-oxidant parameters are shown in Fig. 5. The lowest MDA, SOD and CAT levels among the groups were measured in the control. VCI_3_ exposure caused important alterations in all investigated biochemical parameter values, depending on the dose. Among the VCI_3_ application groups, the highest rate of these increases was detected in 200 µg/L VCI_3_ treated group. While MDA, an indicator of lipid peroxidation, was 6.5 µM/g in the control, VCI_3_ administration increased the MDA level significantly. The most significant increase was observed in the 200 µg/L VCI_3_-treated group, and the MDA level in this group increased by 65.4% compared to the control and was measured as 18.8 µM/g. Lipid peroxidation is an indicator of oxidative stress, and antioxidant enzyme activities were also induced against increased oxidative stress. In Group IV, when compared with the control, SOD activity increased 1.9 times and CAT activity increased approximately 2.7 times. Similarly, Abedini et al.^46^ reported that MDA levels increased in sunflower plant exposed to ammonium metavanadate (NH_4_VO_3_) at 3.25, 7.5 and 15 mg/L doses, and also antioxidant enzyme activities enhanced. Imtiaz et al.^47^ observed that SOD and CAT activities increased linearly with increasing NH_4_VO_3_ concentrations in *Cicer arietinum* L. (chickpea) when grown in vanadium-contaminated soil. Altaf et al.^48^ observed that NH_4_VO_3_ exposure for two weeks caused an increase in enzyme activities and MDA levels of pepper seedlings at all dose levels.

MDA is a three-carbon dialdehyde and formed by the peroxidation of lipids in the cell membrane. In other words, MDA is a reliable indicator for assessing cell damage due to lipid peroxidation^49^. Studies have shown that MDA can alter DNA and RNA, cause cross-links in proteins and lipids, and suppress many genes involved in the plant's response to stressors. It is also known that MDA is carcinogenic and mutagenic^50^. One of the most important effects of metal toxicity is the production of ROS and its ability to induce irreversible damage to macromolecules such as DNA and protein in plants. Plants resort to antioxidant defense system enzymes to scavenge the produced ROS. Two of the most important of these enzymes are SOD and CAT^51^. In this study, the increase in MDA levels and SOD and CAT enzyme activity as a result of VCI_3_ exposure may be due to the increased free radical levels in the cell. H_2_O_2_ is the main component of these free radicals and is produced in excess when plants are exposed to heavy metals. Produced these free radicals can also cause membrane damage in the roots and increase lipid destruction, which leads to MDA production^46,47^. In order to defend itself against these damages of free radicals, the plant activates antioxidant enzyme genes and increases antioxidant enzyme synthesis. The findings of some studies on vanadium toxicity in the literature also support this idea. In the literature, it has been reported that H_2_O_2_ production in the cell increases as a result of exposure to vanadium in different plant species. This increase enhances the lipid peroxidation and oxidative damage, and as a result, antioxidant enzyme activities increase linearly as a cellular defense mechanism^47,48^.

### Anatomical findings

**Figure 6:**
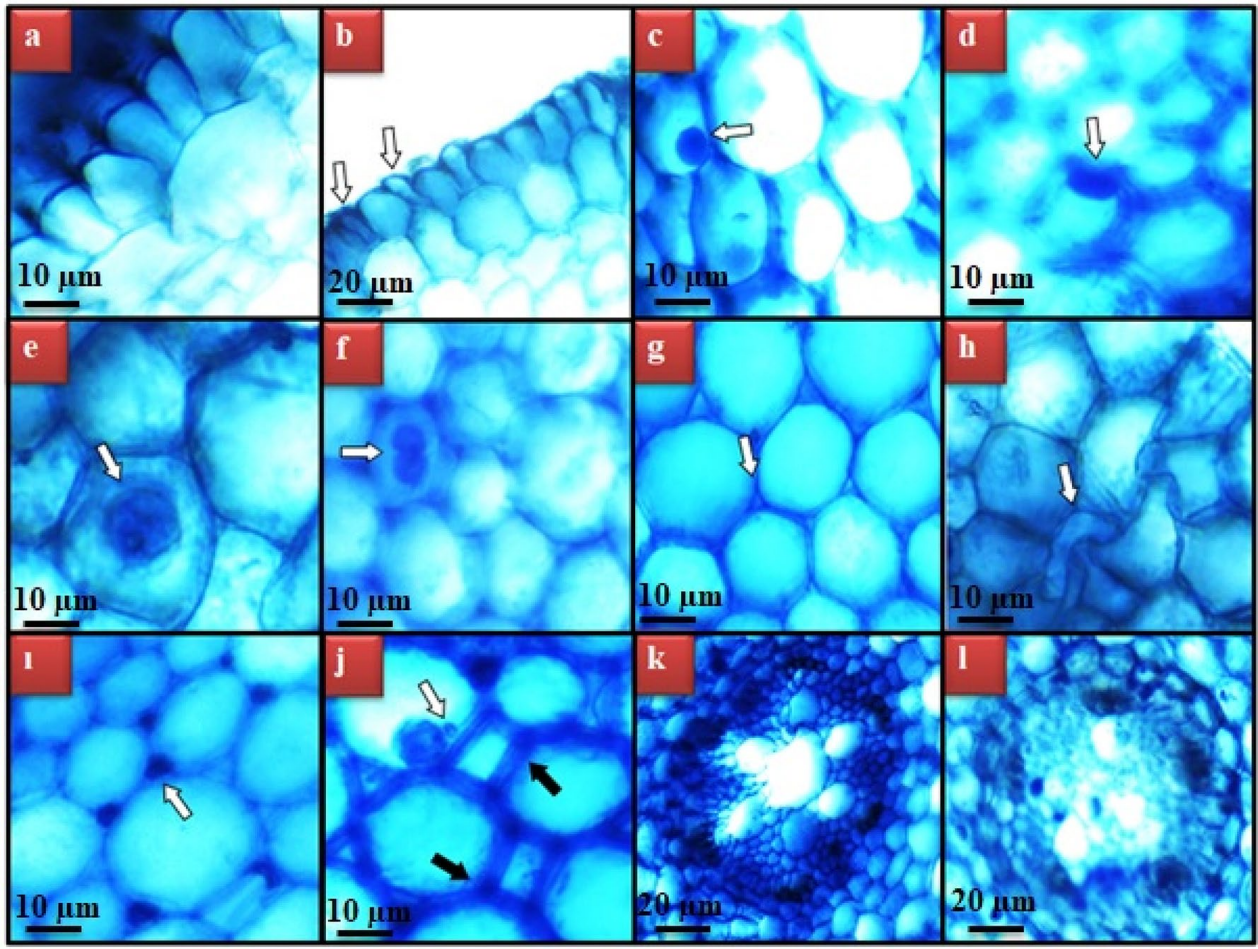
Meristematic cell damages induced by VCI_3_ toxicity. The appearance of epidermis cells in control (**a**), damages in epidermis cell (**b**), cell nucleus (oval) of control (**c**), flattened cell nucleus (**d**), giant nucleus (**e**), binuclear cell (**f**), cortex cells in control (**g**), damages in cortex cell (**h**), cell wall thickening (**i**), MN-white arrow, cell wall thickening-black arrow (**j**), vascular tissue in control (**k**), unclear vascular tissue (**l**).

The damages induced by VCI_3_ exposure and the severities of these damages are given in Table 3. VCI_3_ exposure caused different types of anatomical damages (Fig. 6) and the severity of the damages increased in a dose-dependent manner. The frequency of anatomical damages varies in each group. While giant nucleus, unclear vascular tissue and binuclear cells were not observed in the 50 µg/L VCI_3_-treated group, flattened cell nucleus, epidermis and cortex cell damages and thickening of the cortex cell wall were detected at a small level. In the group in which 200 µg/L VCl_3_ was applied, unclear vascular tissue and binuclear cells were detected at a moderate level, while other abnormalities were detected at severe level. In the literature, the anatomical changes induced by vanadium compounds in the roots of different plant species have been discussed at the macroscopic or morphological level rather than the cellular level. Therefore, the number of studies at the cellular level is extremely small. In one of these studies carried out by our study team, Macar et al.^14^ have reported that exposure to vanadium sulfate pentahydrate at 25, 50 and 100 µg/L doses caused cellular damages such as epidermis cell damage and enlargement of the cell nucleus volume in meristem cells. In another study, Li et al.^52^ observed that vanadium dioxide nanoparticles damaged the root structure by causing shrinkage in xylem conduction tissue cells in pea seedlings. In the study performed at the macroscopic level, Vachirapatama et al.^35^ found that exposure to NH_4_VO_3_ in the 20–80 mg/L dose range caused the darkening of tomato roots and disrupted the root structure.

**Table 3:** The severity of damages induced by VCI_3_ in root meristem cells.

	ECD	FCN	GN	BC	CCD	CCWT	UVT
Control	N.O	N.O	N.O	N.O	N.O	N.O	N.O
50 µg/L VCI_3_	**+**	**+**	N.O	N.O	**+**	**+**	N.O
100 µg/L VCI_3_	**++**	**++**	**+**	**+**	**++**	**++**	**+**
200 µg/L VCI_3_	**+++**	**+++**	**++**	**++**	**+++**	**+++**	**++**

*CCD* cortex cell damage, *CCWT* cortex cell wall thickening, *UVT* unclear vascular tissue, *FCN* flattened cell nucleus, *GN* giant nucleus, *BC* binuclear cell, *ECD* epidermis cell damage. *N.O* not observed. (+++): severe damage, (++): moderate damage, (+): little damage, (*−*): no damage.

The biotransformation process including reduction reactions takes place in the uptake of vanadium by plant roots, and these reactions provide strong attachment and immobilization of vanadium to root tissues. In this way, vanadium remaining in the root for a long time causes serious anatomical effects^5^. As a result of VCI_3_ treatment, the cell deformation, flattened nucleus, giant nucleus and binuclear cell abnormalities observed in root meristem cells show that vanadium remains in the cell and causes abnormalities, especially in the nucleus. The increase in MDA level induced by vanadium indicates an increase in lipid peroxidation and oxidative stress, and DNA fragmentation indicates deterioration in DNA integrity. These damages make the changes in the dynamics of nucleus inevitable and shape changes may occur in the nucleus, which is spherical or ellipsoidal under normal conditions^19,20^. A significant increase in the number of epidermis cells was determined in the groups exposed to VCI_3_ and these cellular increases act as a barrier that reduces the entry of vanadium into the cell. These increases also increase the contact of cells with each other and deformations may occur in the nuclei of the cells and these cells due to formed mechanical pressure. Thickening of the cortex cell wall associated with the accumulation of structures such as cellulose and suberin to provide support and strength to the cells is a defense mechanism that the plant develops directly to reduce vanadium entry^53^. While unclear vascular tissue was not observed in the 50 µg/L VCI_3_-treated group, it was observed at a low level in the 100 µg/L VCI_3_ and moderate level in the 200 µg/L VCI_3_-treated group. With the mechanisms developed by the plant such as thickening of the cortex cell wall and tightening of the epidermis cells the vanadium was prevented from reaching the conductive tissue, therefore, no vascular tissue damage was observed at a dose of 50 µg/L. However, with the increase in dose, the tolerance mechanism was insufficient and damages were observed in the vascular tissue in 100 µg/L and 200 µg/L VCI_3_ applications. This uncertainty in the vascular tissue can be explained by the fragmentation of cell membranes, accumulation of materials or necrotic regions^54,55^.

### Correlation and principal component analysis of parameters

**Figure 7:**
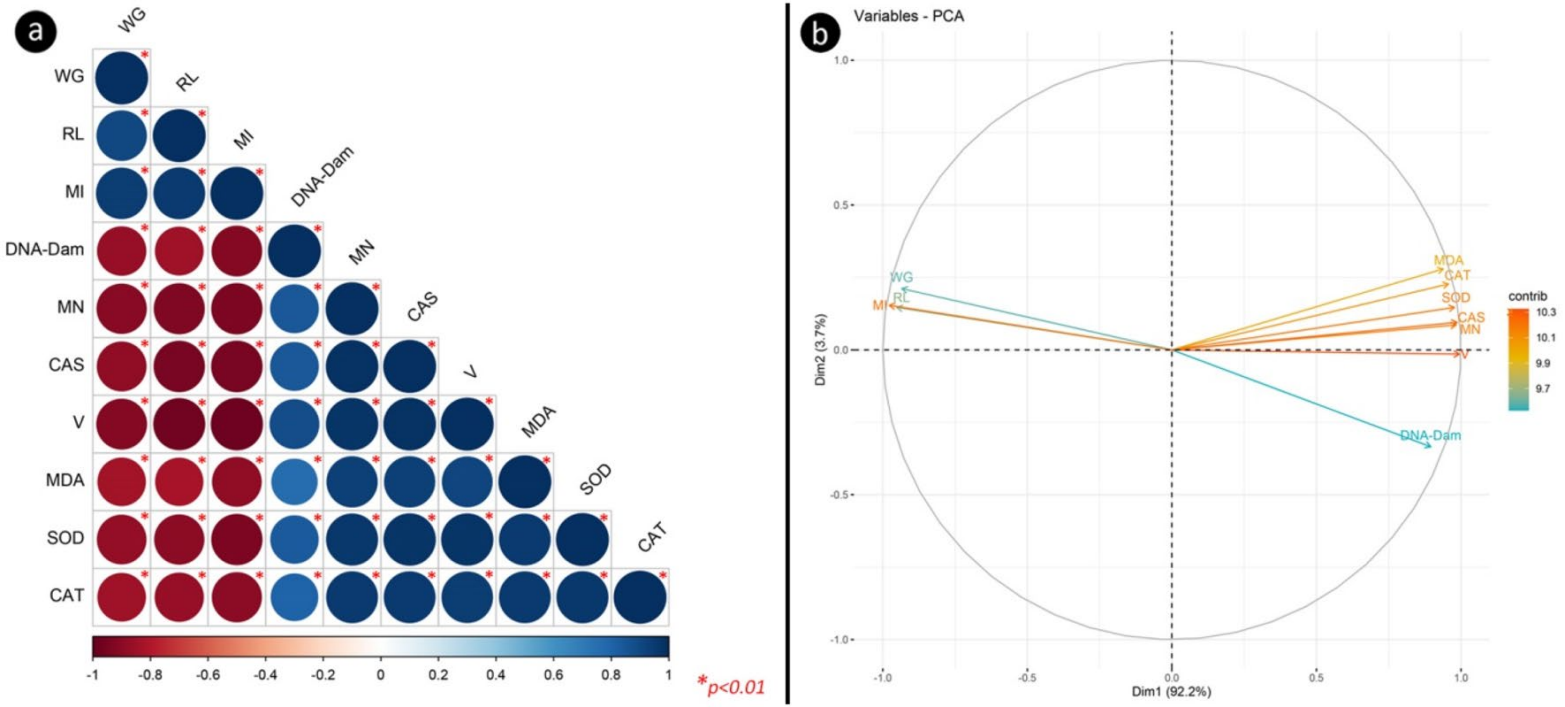
Correlation and principal component analyses (*PCA*) of all investigated parameters. Correlation of all physiological, biochemical and genetic parameters and VCI_3_ application (**a**), PCA analysis of all parameters (**b**). *RL* root length, *WG* weight gain, *DNA-dam* DNA damage score, *V* vanadium.

Correlation analysis of the investigated physiological, cytogenetic and biochemical parameters is shown in Fig. 7a. Positive correlations are expressed in blue, while negative correlations are represented in red. Color intensity and circle size are given in proportion to the correlation coefficients. When the correlation data were examined, it was determined that all parameters had significant positive or negative effects on each other. VCI_3_ exposure was negatively correlated with weight gain, root length and MI, and positively correlated with DNA damage, MN frequency, CAs number, MDA, SOD and CAT levels. In addition, weight gain, root length, MI index parameters, among the parameters examined in the study, showed negative correlations with MN frequency, DNA damage score, CAs number, VCI_3_ dose, and MDA, SOD and CAT levels. In the current study, the statistical data reduction method PCA was used to reduce the complexity of data interpretation in multiple biomarkers analyzes and is given in Fig. 4b. PCA analysis provided a more reliable and complete understanding of the toxicity status and the relationships between biomarkers. Relationships between applied VCI_3_ doses and two physiological, three biochemical and four genetic parameters were investigated using PCA analysis. In Fig. 7b, the two dimensions of the biplot together explain 95.9% of the overall variance. While the contribution of the first axis (*dim1*) to the variance is 3.7%, the second axis (*dim2*) explains most of the variance with 92.2%. It was seen that the contributions of MDA, SOD and CAT components to dim1 and dim2 were quite close to each other. It is seen that these parameters have a very positive component in the dim1 axis and a mild-moderate positive component in the dim2 axis. It is seen that the contribution of dim1 and dim2 axes in CAs and MN levels is very close to each other; these parameters are very positive component in dim1 and slightly positive component in dim2. It was observed that the contribution of VCI_3_ to the dim1 axis is high and there is a very positive component in this axis, and a very slight negative component in the dim2 axis. It was observed that the contribution of MI and RL levels to the dim1 and dim2 axes were quite close to each other, while the WG was partially close. WG, RL, and MI have a very negative component in the dim1 axis and a slightly positive component in the dim2 axis. PCA analyzes confirm the interrelationships of VCI3 exposure in the physiological, cytogenetic, and biochemical parameters tested.

## Conclusion

The excessive use of petroleum products, especially in industry and transportation, pesticide and fertilizer applications containing vanadium in agriculture cause excessive accumulation of vanadium in the air, water and especially in the soil. This contamination can reach all living things through the food chain and cause various toxic effects. In this study, it was determined that vanadium chloride caused a wide-ranging toxic effect on *A. cepa*, a bio-indicator organism. Vanadium exposure caused a decline in physiological parameters related to plant growth. One possible reason for this regression is the decrease in the mitotic index, and there were significant reductions in dividing cell numbers in the vanadium-treated groups compared to the control. These reductions may be associated with the formation of CAs and MN induced by vanadium. Such genome instabilities cause cell cycle delays and a direct decrease in cell proliferation. Vanadium exposure also caused deterioration in the antioxidant/oxidant balance in stem cells, and the increase in MDA level and the increase in antioxidant enzyme activities indicate oxidative stress. The main reason for the physiological regressions and cyto-genotoxic effects is the oxidative stress induced by vanadium. The cell has developed mechanisms to cope with oxidative stress and to develop tolerance to vanadium, such as induction of antioxidant enzymes and alteration of various anatomical structures. Since this study is the most comprehensive study that deals with the toxicity of VCI_3_ in plants with the help of many different parameters and provides information about the possible mechanism of toxicity, it provides important contributions to the literature and serves as a guide for future studies.

## Data Availability

The datasets used and/or analyzed during the current study are available from the corresponding author on reasonable request.

## References

[1] Osamu WADA (2004). What are trace elements?. JMAJ..

[2] Al-Fartusie FS, Mohssan SN (2017). Essential trace elements and their vital roles in human body. Indian J. Adv. Chem. Sci..

[3] Mwakikunga, B. W. Vanadium metal and compounds, properties, interactions, and applications. In *Encyclopedia of Metalloproteins* (eds. Kretsinger, R. H., Uversky, V. N. & Permyakov, E.A.) 2316–2324 (New York, 2013).

[4] Sutradhar, M., Da Silva, J. A. L. & Pombeiro, A. J. L. Chapter 1: Introduction: Vanadium, its compounds and applications. In *Vanadium Catalysis* 1–11 (2020).

[5] Roychoudhury A (2020). Vanadium uptake and toxicity in plants. SF J. Agric. Crop Manag..

[6] Pourret, O., Dia, A. Vanadium. In *Encyclopedia of Geochemistry* (ed. White, W. M.), 1–3 (Switzerland, 2016).

[7] Chen L, Liu JR, Hu WF, Gao J, Yang JY (2021). Vanadium in soil-plant system: Source, fate, toxicity, and bioremediation. J. Hazard. Mater..

[8] Waters, M. D. Toxicology of vanadium. İn *Advances in Modern Toxicology* (ed. Goyer, R. A. & Mehlman, M. A.) 147–189 (New York, 1977).

[9] Marczewski K, Marczewska B, Kuzioła R (2015). The Importance of vanadium concentration in ground and deep ground water for spring water quality. BCPM.

[10] Bishayee A, Waghray A, Patel MA, Chatterjee M (2010). Vanadium in the detection, prevention and treatment of cancer: The in vivo evidence. Cancer Lett..

[11] Garau G (2015). Detoxification processes from vanadate at the root apoplasm activated by caffeic and polygalacturonic acids. PLoS ONE.

[12] Lin CY, Trinh NN, Lin CW, Huang HJ (2013). Transcriptome analysis of phytohormone, transporters and signaling pathways in response to vanadium stress in rice roots. Plant Physiol. Biochem..

[13] Imtiaz M (2016). Comparison of antioxidant enzyme activities and DNA damage in chickpea (*Cicer arietinum* L.) genotypes exposed to vanadium. Environ. Sci. Pollut. Res..

[14] Macar, O., Macar, T. K., Yalçın, E. & Çavuşoğlu, K. Investigation of the physiological and anatomical effects of vanadium (V) toxicity in *Allium cepa* L. Black Sea 1st International Multidisciplinary Scientific Studies Congress, 158–162, Giresun-Turkiye (2019).

[15] Yalçın E, Çavuşoğlu K (2022). Toxicity assessment of potassium bromate and the remedial role of grape seed extract. Sci. Rep..

[16] Demirtaş G, Çavuşoğlu K, Yalçin E (2020). Aneugenic, clastogenic, and multi-toxic effects of diethyl phthalate exposure. Environ. Sci. Pollut. Res..

[17] Türkoğlu Ş (2012). Determination of genotoxic effects of chlorfenvinphos and fenbuconazole in *Allium cepa* root cells by mitotic activity, chromosome aberration, DNA content, and comet assay. Pestic. Biochem. Physiol..

[18] Yalçın E, Çavuşoğlu K (2022). Spectroscopic contribution to glyphosate toxicity profile and the remedial effects of *Momordica charantia*. Sci. Rep..

[19] Tümer C, Çavuşoğlu K, Yalçin E (2022). Screening the toxicity profile and genotoxicity mechanism of excess manganese confirmed by spectral shift. Sci. Rep..

[20] Aydın D, Yalçın E, Çavuşoğlu K (2022). Metal chelating and anti-radical activity of *Salvia ofcinalis* in the ameliorative effects against uranium toxicity. Sci. Rep..

[21] Macar O, Kalefetoğlu Macar T, Yalçın E, Çavuşoğlu K (2022). Acute multiple toxic efects of trifoxystrobin fungicide on *Allium cepa* L. Sci. Rep..

[22] Fenech M (2003). HUMN project: Detailed description of the scoring criteria for the cytokinesis-block micronucleus assay using isolated human lymphocyte cultures. Mutat. Res..

[23] Chakraborty R, Mukherjee AK, Mukherjee A (2009). Evaluation of genotoxicity of coal fly ash in *Allium cepa* root cells by combining comet assay with the *Allium* test. Environ. Monit. Assess..

[24] Końca K (2003). A cross-platform public domain PC image-analysis program for the comet assay. Mutat. Res..

[25] Collins AR (2004). The comet assay for DNA damage and repair. Mol. Biotechnol..

[26] Unyayar S, Celik A, Cekic FO, Gozel A (2006). Cadmium-induced genotoxicity, cytotoxicity and lipid peroxidation in *Allium sativum* and *Vicia faba*. Mutagenesis.

[27] Zou J, Yue J, Jiang W, Liu D (2012). Effects of cadmium stress on root tip cells and some physiological indexes in *Allium cepa* var. agrogarum L. Acta Biol. Cracov. Bot..

[28] Beauchamp C, Fridovich I (1971). Superoxide dismutase: Improved assays and an assay applicable to acrylamide gels. Anal. Biochem..

[29] Beers RF, Sizer IW (1952). Colorimetric method for estimation of catalase. J. Biol. Chem..

[30] Çavuşoğlu K, Kurt D, Yalçın E (2020). A versatile model for investigating the protective effects of *Ceratonia siliqua* pod extract against 1,4-dioxane toxicity. Environ. Sci. Pollut. Res..

[31] RStudio Team. RStudio: integrated development for R. RStudio, PBC, Boston, MA. http://www.rstudio.com/ (2021).

[32] Wei T (2017). Package ‘corrplot’. Statistician.

[33] Lê S, Josse J, Husson F (2008). FactoMineR: An R package for multivariate analysis. J. Stat. Softw..

[34] Kassambara, A. & Mundt, F. Factoextra: extract and visualize the results of multivariate data analysis. R package version 1.0.5. https://CRAN.R-project.org/package=factoextra (2017).

[35] Vachirapatama N, Jirakiattiku Y, Dicinoski GW, Townsend AT, Haddad PR (2011). Effect of vanadium on plant growth and its accumulation in plant tissues. Songklanakarin J. Sci. Technol..

[36] Aihemaitia A (2019). The effect of vanadium on essential element uptake of *Setaria viridis*’ seedlings. J. Environ. Manag..

[37] Sipahi Kuloğlu S, Yalçın E, Çavuşoğlu K, Acar A (2022). Dose-dependent toxicity profile and genotoxicity mechanism of lithium carbonate. Sci. Rep..

[38] Marcano L, Carruyo I, Fernández Y, Montiel X, Torrealba Z (2006). Determination of vanadium accumulation in onion root cells (*Allium cepa* L.) and its correlation with toxicity. Biocell.

[39] Rodríguez-Mercado, J. J., Roldán-Reyes, E. & Altamirano-Lozano, M. (2003). Genotoxic effects of vanadium (IV) in human peripheral blood cells. Toxicol. Lett..

[40] Rodríguez-Mercado JJ, Álvarez-Barrera L, Altamirano-Lozano MA (2010). Chromosomal damage induced by vanadium oxides in human peripheral lymphocytes. Drug Chem. Toxicol..

[41] Çanlı M (2018). A new perspective to aberrations caused by barium and vanadium ions on *Lens culinaris* Medik. Ecotox. Environ. Safe..

[42] Seth CS, Misra V, Chauhan LKS, Singh RR (2008). Genotoxicity of cadmium on root meristem cells of *Allium cepa*: Cytogenetic and comet assay approach. Ecotoxicol. Environ. Saf..

[43] Yıldız M, Ciğerci İH, Konuk M, Fidan AF, Terzi H (2009). Determination of genotoxic effects of copper sulphate and cobalt chloride in *Allium cepa* root cells by chromosome aberration and comet assays. Chemosphere.

[44] Leopardi P (2005). Assessment of the in vivo genotoxicity of vanadate: Analysis of micronuclei and DNA damage induced in mice by oral exposure. Toxicol. Lett..

[45] Rivas-García L (2020). In vitro study of the protective effect of manganese against vanadium-mediated nuclear and mitochondrial DNA damage. Food Chem. Toxicol..

[46] Abedini M, Fatemeh Mohammadian FM, Daie-Hassani B, Zarandi-Miandoab L (2017). Antioxidant responses of *Helianthus annuus* L. under vanadium stress. Iran. J. Plant Physiol..

[47] Imtiaz M (2018). Vanadium toxicity in chickpea (*Cicer arietinum* L.) grown in red soil: Effects on cell death, ROS and antioxidative systems. Ecotox. Environ. Safe..

[48] Altaf MA (2021). Vanadium toxicity induced changes in growth, antioxidant profiling, and vanadium uptake in pepper (*Capsicum annum* L.) seedlings. Horticulturae.

[49] Dikker O, Şahin M, Atar S, Bekpınar S (2018). Examination of oxidative stress markers in women with postmenopausal osteoporosis. Turk. J. Osteoporos..

[50] Davey MW, Stals E, Panis B, Keulemans J, Swennen RL (2005). High-throughput determination of malondialdehyde in plant tissues. Anal. Biochem..

[51] Ighodaro OM, Akinloye OA (2018). First line defence antioxidants-superoxide dismutase (SOD), catalase (CAT) and glutathione peroxidase (GPX): Their fundamental role in the entire antioxidant defence grid. Alexandria J. Med..

[52] Li Q (2022). Phytotoxicity of VO_2_ nanoparticles with different sizes to pea seedlings. Ecotox. Environ. Safe..

[53] Çavuşoğlu K, Yalçın E (2023). Spectral shift supported epichlorohydrin toxicity and the protective role of sage. Environ. Sci. Pollut. Res..

[54] Mithöfer, A. & Maffei, M. E. General mechanisms of plant defense and plant toxins. *Plant Toxins* 3–24 (2017).

[55] Doğan M, Çavuşoğlu K, Yalçın E, Acar A (2022). Comprehensive toxicity screening of pazarsuyu stream water containing heavy metals and protective role of lycopene. Sci. Rep..

